# Rare Genetic Developmental Disabilities: Mabry Syndrome (MIM 239300) Index Cases and Glycophosphatidylinositol (GPI) Disorders

**DOI:** 10.3390/genes15050619

**Published:** 2024-05-14

**Authors:** Miles D. Thompson, Alexej Knaus

**Affiliations:** 1 Krembil Brain Institute, Toronto Western Hospital, 399 Bathurst Street, Toronto, ON M5T 2S8, Canada; 2 Institute for Genomic Statistics and Bioinformatics, University Hospital Bonn, University of Bonn, 53127 Bonn, Germany; knausa@uni-bonn.de

**Keywords:** case study, hyperphosphatasia with neurologic deficit (HPMRS), Mabry syndrome, glycophosphatidylinositol (GPI) biosynthesis disorder (GPIBD), whole exon sequencing, identity by descent filtering, whole genome sequencing, human phenotype ontology (HPO) analysis

## Abstract

The case report by Mabry et al. (1970) of a family with four children with elevated tissue non-specific alkaline phosphatase, seizures and profound developmental disability, became the basis for phenotyping children with the features that became known as Mabry syndrome. Aside from improvements in the services available to patients and families, however, the diagnosis and treatment of this, and many other developmental disabilities, did not change significantly until the advent of massively parallel sequencing. As more patients with features of the Mabry syndrome were identified, exome and genome sequencing were used to identify the glycophosphatidylinositol (GPI) biosynthesis disorders (GPIBDs) as a group of congenital disorders of glycosylation (CDG). Biallelic variants of the phosphatidylinositol glycan (PIG) biosynthesis, type V (*PIGV*) gene identified in Mabry syndrome became evidence of the first in a phenotypic series that is numbered HPMRS1-6 in the order of discovery. HPMRS1 [MIM: 239300] is the phenotype resulting from inheritance of biallelic *PIGV* variants. Similarly, HPMRS2 (MIM 614749), HPMRS5 (MIM 616025) and HPMRS6 (MIM 616809) result from disruption of the *PIGO*, *PIGW* and *PIGY* genes expressed in the endoplasmic reticulum. By contrast, HPMRS3 (MIM 614207) and HPMRS4 (MIM 615716) result from disruption of post attachment to proteins *PGAP2* (HPMRS3) and *PGAP3* (HPMRS4). The GPI biosynthesis disorders (GPIBDs) are currently numbered GPIBD1-21. Working with Dr. Mabry, in 2020, we were able to use improved laboratory diagnostics to complete the molecular diagnosis of patients he had originally described in 1970. We identified biallelic variants of the *PGAP2* gene in the first reported HPMRS patients. We discuss the longevity of the Mabry syndrome index patients in the context of the utility of pyridoxine treatment of seizures and evidence for putative glycolipid storage in patients with HPMRS3. From the perspective of the laboratory innovations made that enabled the identification of the HPMRS phenotype in Dr. Mabry’s patients, the need for treatment innovations that will benefit patients and families affected by developmental disabilities is clear.

## 1. Introduction

The use of targeted panel whole exome sequencing (WES), whole genome sequencing (WGS), and increasingly, RNA sequencing (RNASeq) combined with WGS, have become the standard for identifying rare genetic developmental disabilities [1,2,3,4,5]. Even with these advances, as many as half of unexplained cases of developmental disability have no known genetic cause [6]. However, this represents a significant improvement from 2010 [7], when the first gene for Mabry syndrome (HPMRS1 or GPIBD2; MIM 239300) was identified by Krawitz et al. [8]. The subsequent advances in genomic diagnostics have had a profound impact on clinical genetics [1,2,3,4,5] and genomic counselling [9]. By these means, novel disease pathways have been identified, including the Glycophosphatidylinositol (GPI) biosynthesis pathway that is disrupted in at least 21 developmental disabilities [10].

Paroxysmal nocturnal hemoglobinuria (PNH), an acquired clonal blood disorder linked to somatic variants of the phosphatidylinositol glycan (PIG) type A (PIGA) gene, was the first disorder known to result from disruption of a GPI gene [11]. However, glycophosphatidylinositol deficiency, resulting from biallelic variants of the *PIGM* gene, encoding the first mannose transferase, was the first inherited phenotype for which germline biallelic variants encoding PIG enzymes were reported [12]. Subsequently, biallelic variants of the gene encoding the second mannose transferase, phosphatidylinositol glycan (PIG) biosynthesis, type V (PIGV), were identified in HPMRS1 [8,13]. Identity by descent (IBD) filtering [8] of data from massively parallel sequencing [14,15,16] enabled this discovery. Since that time, laboratory diagnosis has become the definitive means of distinguishing rare developmental disabilities in many cases. This has been facilitated by the number of exomes and genomes available for comparison (https://gnomad.broadinstitute.org/, accessed on 7 April 2024) as well as improved in silico prediction tools available for the analysis of likely pathogenic variants [17].

HPMRS is a genetically heterogeneous syndrome. Conventional genetic testing, including gene-based or panel-based methods, may miss pathogenic variants. Increasingly, WGS combined with RNAseq may be preferable to panel sequencing or WES to identify intronic and 3′UTR variants, since the latter may miss non-coding variants [1,2,3,4,5]. The advances made possible by improved variant detection and interpretation create pharmacogenomic opportunities for precision medicine to target an individual’s unique, rare variant [18,19].

### Phenotyping

**Figure 1:**
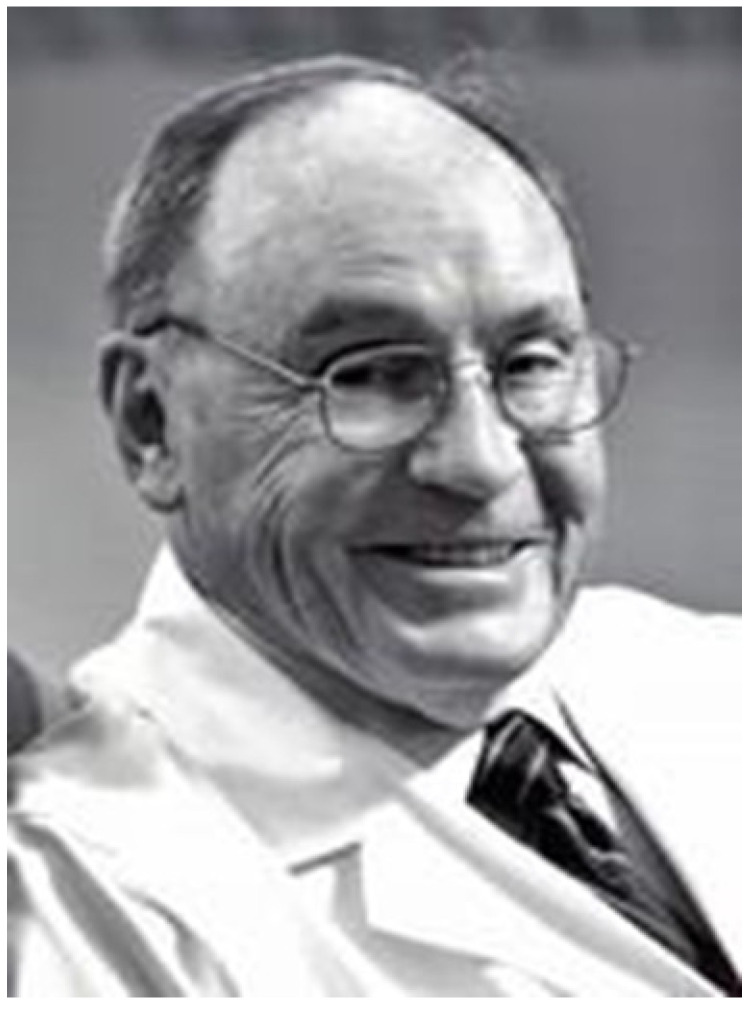
C. Carleton Mabry (1930–2021). Reproduced with permission from David Mabry.

The progress in neurogenetics, developmental disability genetics and genetic counselling of rare diseases can be illustrated by the fact that the 50th anniversary of the publication by Mabry et al. [20] of the case report, ‘Familial hyperphosphatasia with mental retardation, seizures, and neurologic deficits (J. Pediatrics. 77 (1), 74–85) was marked by the identification of likely pathogenic variants in those index patients [21]. This report of a family with four children who experienced a profound developmental disability provided the prototypic features of a rare disorder [22,23,24,25,26,27] that enabled the discovery of a novel class of glycophosphatidylinositol (GPI) disorders [8]. Dr. C. Charlton Mabry (Figure 1) phenotyped the patients at the University of Kentucky School of Medicine. His work became a significant resource for those who subsequently diagnosed and reported the syndrome [22,23,24,25]. This led to case series [26,27] and the proposal that the syndrome be named Mabry syndrome (MIM 239300): https://www.youtube.com/watch?v=PlE0U0tRh3E, accessed on 7 April 2024.

## 2. Genetic Heterogeneity of Mabry Syndrome

As more patients with the Mabry syndrome triad of hypophosphatasia, seizures and developmental disability [11,20,21,22,23,24,25,26,27] were identified and more exomes analyzed, the clinical and genetic heterogeneity of HPMRS became evident [28]; along with the fact that the majority of GPIBDs do not demonstrate hyperphosphatasia [29,30,31,32]. For example, the majority of patients with multiple congenital anomalies hypotonia and seizures, type 1 (MCAHS1; MIM 614080) and Fryn syndrome (MIM 229850) [33,34,35], resulting from biallelic *PIGN* variants, do not demonstrate hyperphosphatasia [31,36].

The HPMRS phenotypes outlined below are numbered HPMRS1-6 in the order that each genotype-phenotype correlation was made; however, due to space limitations, the references cited in the text are generally limited to the first reports implicating a specific gene in HPMRS. The nomenclature itemizing all 21 GPIBDs reflects the order in which the gene responsible for each was reported in the context of other GPIBDs [29,30,31]. As a group of related disorders, the GPIBDs may represent 0.15% of those with developmental disability, according to the Deciphering Developmental Disorders (DDD) study [37].

In addition to the *PIGV* variants identified in HPMRS1 [8], pathogenic variants of at least three additional PIG genes expressed in the endoplasmic reticulum (ER) are also reported to result in Mabry syndrome. HPMRS2 [MIM: 614749], or GPIBD6, was reported to result from biallelic variants of the gene encoding *PIGO*, the phosphoethanolamine transferase that transfers phosphoethanolamine to the third mannose [38,39,40]. HPMRS5 [MIM: 616025], or GPIBD11, was reported to result from biallelic variants of *PIGW*, the gene encoding an inositol acyltransferase that acylates the inositol ring of phosphatidylinositol [41]. HPMRS6 [MIM: 616809], or GPIBD12, was reported to result from biallelic variants of *PIGY*, a gene that encodes a component of the GPI-N-acetylglucosaminyltransferase (GIP-GnT) complex which initiates biosynthesis [42]. Hyperphosphatasia has also been reported in at least some patients with other GPIBDs, including multiple congenital anomalies hypotonia and seizures, type 2 (MCAHS2; MIM 300868) and coloboma, congenital heart disease, ichthyosiform dermatosis, impaired intellectual development, and ear anomalies (CHIME; MIM 280000) syndrome. MCAHS2, results from biallelic variants in *PIGA,* which encodes an enzyme involved in synthesis of N-acetylglucosaminyl phosphatidylinositol (GlcNAc-PI), the first intermediate of the GPI anchor [43]. CHIME syndrome results from biallelic variants in *PIGL*, a gene encoding the enzyme catalyzing the second glycosylphosphatidylinositol (GPI) biosynthesis step, de-N-acetylation of N-acetylglucosaminylphosphatidylinositol (GlcNAc-PI) [44]. It should be noted, however, that a GPIBD only meets the criteria for HPMRS if there is a persistent, stable elevation of alkaline phosphatase [18,26,27].

In addition to the HPMRS phenotypes attributed to hypomorphic variants of PIG biosynthesis enzymes expressed in the ER, two phenotypes have been reported to result from variants of genes encoding enzymes expressed in the Golgi. Biallelic variants have been reported in two genes encoding post-GPI attachment to proteins (PGAP) enzymes that act in the Golgi to stabilize membrane attachment of GPI-anchored proteins (GPI-AP). HPMRS3 [MIM: 614207], or GPIBD8, has been associated with biallelic variants of *PGAP2*, a gene encoding a membrane protein required, during fatty-acid remodeling, for reacylation of the GPI-lyso intermediate with a saturated fatty acid, such as stearic acid [45,46,47,48]. HPMRS4 [MIM: 615716], or GPIBD10, has been associated with biallelic variants of *PGAP3*, the gene encoding a seven transmembrane protein that removes an unsaturated fatty acid from the sn-2 position of GPI prior to reacetylation by PGAP2 [49,50]. 

While disruption to the transfer of the nascent peptide to the GPI anchor by the transamidase complex is not commonly associated with hyperphosphatasia [13], the central pathogenic mechanism of HPMRS may be associated with a defect in ER-Golgi transport. Like Wolfram syndrome I (WFS1) [51], ER-Golgi transit defects may underlie HPMRS; although, the fact that they share few phenotypic features [13,51], suggests the distinct pathways that are involved.

### Mabry Syndrome Index Cases

**Figure 2:**
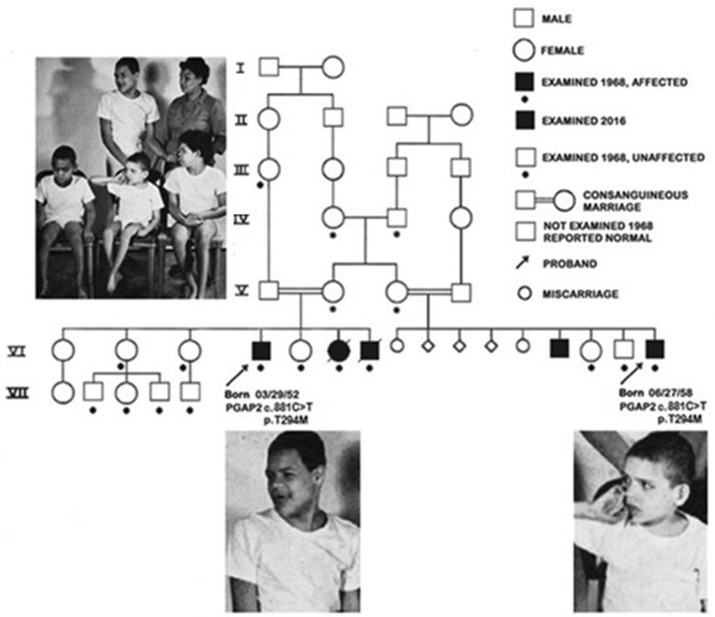
The original Mabry syndrome index cases, front row, from left: VI-7 (born. 02/17/57; died 05/31/71), VI-16 (born 06/27/58), and VI-6 (born 10/30/55; died 10/19/85); back row: VI-4 (born 03/29/52) and normal mother (V-2) of VI-4, -6 and -7. Patient VI-4 and patient VI-16, photographed in Lexingon, Kentucky, were both homozygous for thec.881C > T, p.T294M *PGAP2* variants. Adapted from Thompson et al., 2020 [30].

As the spectrum of HPMRS phenotypes became evident, however, a molecular diagnosis of Dr. Mabry’s original patients seemed elusive, since they had been lost to follow-up many decades previously. Beginning in 2008, we proposed using emerging technologies to identify the pathogenic variant in the Mabry syndrome index cases. However, it can be challenging to update records that predate the electronic era. The original case report was based on information collected as part of a mobile genetics unit from University of Kentucky that visited neighboring Tennessee [20,21]. This, in itself, reflects the challenges that families with developmentally disabled children faced in early days of Medicaid. Although the records of the visits were easily identified, since the patients (Figure 2) were admitted to University of Kentucky on 5 November 1968, follow-up was initially not possible.

**Figure 3:**
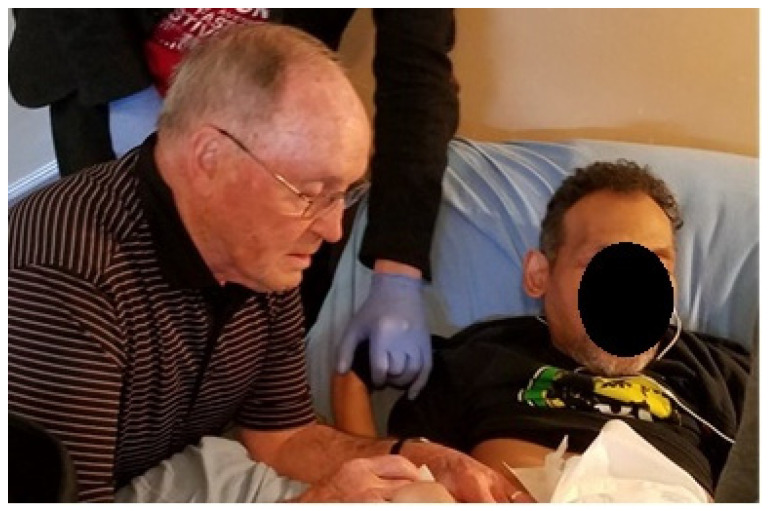
Dr. Mabry working with a patient. Reproduced with permission from David Mabry.

Despite extensive searches, it was not until 2016, that Dr. Mabry located the surviving patients. With support from the University of Kentucky, we obtained informed consent to visit the two surviving patients in their respective group homes in rural Appalachia [51]. This resulted in a field trip in which we visited the original Mabry syndrome patients at their respective group homes. Figure 2 shows the appearance of these first cousins when originally examined in 1968 in the context of the pedigree. We conducted neurological evaluation and obtained samples for biochemical and genomic analysis [21]. Figure 3 shows Dr. Mabry working with a patient.

Examination of patient 1-VI-4 (born 03/29/52) and 1-VI-16 (born 06/27/58) allowed the evaluation of the natural history of the disorder after nearly 50 years. Alkaline phosphatase levels remained elevated over the course of their lives. During adult-hood, however, 1-VI-4 and 1-VI-16 no longer experienced spontaneous seizures and were no longer administered anticonvulsants. Unfortunately, the age at which this occurred appears not to have been documented [51].

We used exome GPI panel sequencing to identify biallelic c.881C > T *PGAP2* variants, expressed as the hypomorphic *PGAP2* c.698C > T, p.Thr233Met isoform 8, that are likely pathogenic in patients with the syndrome Dr. Mabry originally reported. We were able to show that the variant *PGAP2* was unable to rescue *PGAP2* deficient cell lines [52]. This evidence indicates that the Mabry syndrome index patients [19] manifested HPMRS3 (GPIBD 8 [MIM: 614207]).

These individuals retain motor control but are hypotonic. It was clear at the time of examination that the phenotype of the two surviving patients has been relatively stable over time, with spontaneous remission of seizures taking place during adulthood. This should be interpreted with caution, however, since two other persons affected in the index family died as a probable result of sudden unexpected death in epilepsy (SUDEP) in 10/19/85 and 05/31/71, despite anticonvulsant medication [21].

## 3. Perspective

The knowledge that Mabry syndrome, results from biallelic inheritance of pathogenic variants that disrupt GPI pathway genes in the biosynthesis stage [29,30,31,32] or the remodeling stage necessary for stable lipid raft association of GPI-APs [53,54,55] now makes it possible to use WGS and/or RNASeq as a first line diagnostic method.

### 3.1. Biosynthesis and Remodeling Phenotypes

**Figure 4:**
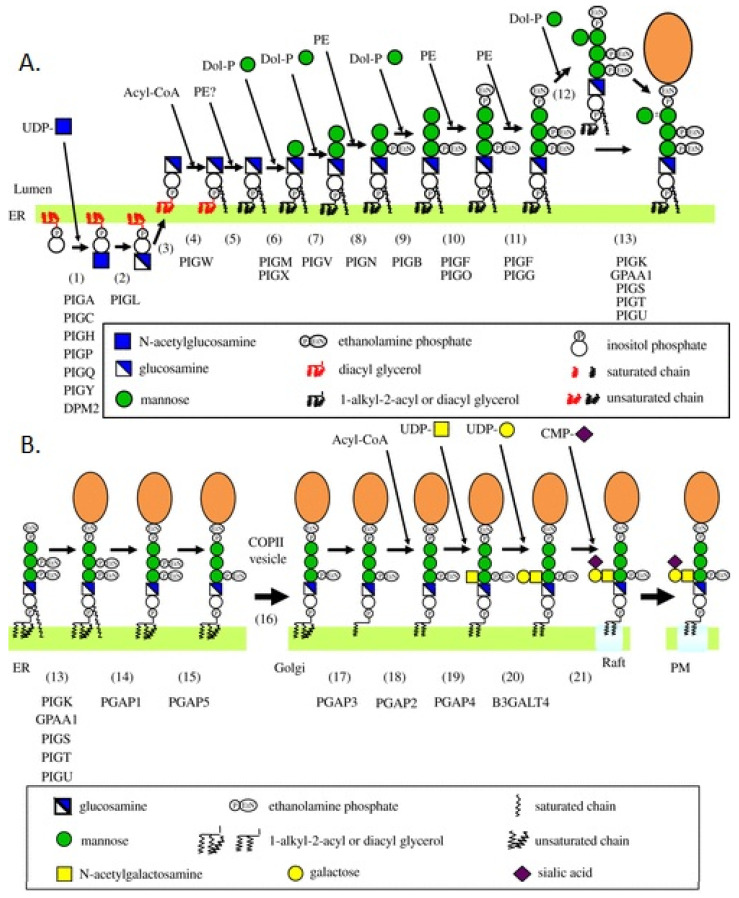
Glycosylphosphatidylinositol biosynthesis, transamidase and remodeling pathways. (**A**) Biosynthesis of mammalian GPI. The complete GPI precursor competent for attachment to proteins is synthesized in the ER from PI by stepwise reactions (1)–(11). Man4 side chain is attached in the ER to some GPI (step (12)). The preassembled GPI is *en bloc* transferred to proteins (step (13)). Genes involved in these reaction steps are shown. (**B**) Maturation of mammalian GPI-APs during ER–plasma membrane (PM) transport. Nascent GPI-APs generated by the transfer of GPIs to proteins (step 12) undergo two reactions, inositol-deacylation (step 14) and removal of the EtNP side chain from Man2 (step 15) in the ER. The ER–Golgi transport of GPI-APs is mediated by COPII-coated vehicles (step 16). In the Golgi apparatus, GPI-APs undergo fatty acid remodeling (steps 17 and 18). Some GPI anchors have a modified GalNAc side chain (steps 19–21). The mature GPI APs are transported to the PM where they are associated with raft microdomains. Genes involved in these reaction steps are shown below step numbers. Adapted from [29]. Reproduced with permission from Kinoshita, T., Open Biology; published by Elsevier, 2020.

Human phenotype ontology (HPO) analysis [53,56] has enabled us to better contrast HPMRS resulting from variants in biosynthesis and remodeling stages [53]: potentially allowing better treatment plans to be developed. HPO analysis, relying upon a standardized vocabulary of phenotypic abnormalities [53], has placed the hyperphosphatasia that distinguishes HPMRS from other GPIBDs [29,30,31] in perspective [53]. In 2020, HPO analysis was used to analyze 58 published cases comprising 152 individual patients with GPIBDs impacting 22 genes. We tested the frequency of HPO terms associated with 1. the stepwise GPI-anchor biosynthesis, and 2. the transamidase and remodeling stage [53], as outlined in Figure 4.

While we found statistically significant differences in the phenotypic spectrum of patients whose disease resulted from disruption of biosynthesis compared with those with disruption in the transamidase and remodeling pathways, skeletal abnormalities were significantly more likely to occur in patients with pathogenic variants in the synthesis stage of the biosynthetic pathway. These patients had a greater occurrence HPO terms (33%) that describe abnormal digit morphology. Moreover, patients in the synthesis group were more likely to have abnormal muscle morphology, tendon morphology, and/or joint morphology compared with the transamidase and remodeling group. By contrast, patients with pathogenic variants disrupting genes active in transamidase and remodeling had fewer incidences (6.7%) of abnormal digit morphology. In particular, remodeling defects manifested diverse phenotypic abnormalities impacting bone and facial development and neurodevelopmental disabilities [53].

### 3.2. Characteristic Facial Gestalt

Facial dysmorphism in patients with GPI anchor deficiency presents a complex array of distinctive features, often serving as a diagnostic hallmark of the condition. Prominent features frequently observed in affected individuals include a broad nasal bridge, hypertelorism, low-set ears, micrognathia (small jaw), and a thin upper lip with a tented appearance and a smooth philtrum. These facial characteristics, among others, are crucial indicators for clinicians in suspecting and diagnosing GPI anchor deficiency.

Automated facial analysis has emerged as an indispensable tool in the accurate diagnosis of genetic conditions and has been applied multiple times in characterization of GPIBD patients. While GPIBDs are considered pathway disorders, there exist facial characteristics that are specific to individual genes. Hence, despite the common assumption of uniformity in appearance, automated facial analysis, for example by GestaltMatcher [57], enables more accurate gene assignment in GPI patients compared to experienced clinicians [31,58,59]. Furthermore, in GPIBD patients, ACMG criterion PP4 (characteristic facial gestalt as a “phenotypic match”) aids in better evaluating variants of unknown clinical significance (VUCs), enabling rapid and accurate diagnosis equivalent to functional characterization through flow cytometry. This approach enhances variant interpretation, ensuring timely and precise diagnostic outcomes.

### 3.3. The Hyperphosphatasia Phenotype

Along with facial dysmorphology and seizures, elevated tissue non-specific alkaline phosphatase is one of the three cardinal signs of Mabry syndrome that typically has onset in the first year or two of life [19]. Using HPO analysis, we reported that hyperphosphatasia, was not strongly associated with either the synthesis or the remodeling phenotypes [53]. Hyperphosphatasia did not excerpt a differential effect on the biochemical disruptions of the GPI pathway that result in different clinical manifestations but was an incidental consequence of disrupting two very different processes that take place in the ER and the Golgi [29,30,53]. This may have relevance to the successful treatment of some HPMRS patients, with pyridoxine hydrochloride (discussed in Section 4.1).

#### 3.3.1. Hyperphosphatasia Resulting from GPI Biosynthesis Disruption

Hypomorphic variants of genes expressed in the ER (*PIGV*, *PIGO*, *PIGW* and *PIGY*) disrupt the stepwise synthesis of the GPI anchor, resulting in an incomplete anchor [8,38,39,40,41,42]. The mechanisms resulting in hyperphosphatasia differ according to the gene and portion of the pathway disrupted. *PIGV* and *PIGO* variants are associated with reduced GPI-AP surface levels and increased extracellular secretion into the extracellular space, resulting in hyperphosphatasia [29,30]. This has been modelled as follows.

The carboxyl terminal of the GPI-attachment signal peptide of the nascent peptide anchors it in the ER membrane. The proximity of the nascent peptide to an incomplete GPI anchor with at least one mannose may result in the recruitment of a portion of the transamidase, PIGU, that directs the catalytic subunit, PIGK, to the cleave the GPI recognition sequence and liberate the non-GPI-anchored, soluble AP [60,61]. Although it has been hypothesized that early biosynthesis defects result in more degradation than secretion of AP, compared with later biosynthesis defects [60,61], the identification of pathogenic variants of *PIGW* and *PIGY* in HPMRS5 and HPMRS6, respectively, may suggest other mechanisms of hyperphosphatasia.

#### 3.3.2. Hyperphosphatasia Resulting from Disruption of Fatty Acid Remodeling

In the remodeling pathway, defects in fatty acid remodeling are known to result from two related disruptions. Pathogenic *PGAP3* variants result in a hypomorphic GPI-specific phospholipase A2 that is unable to deacetylate the 2-acyl unsaturated fatty acid. This results in an unstable anchor, incompatible with lipid raft association, which is either released from a cell or degraded. The unsaturated fatty acid is cleaved by phospholipase C [49,50], resulting in hyperphosphatasia. By contrast, pathogenic *PGAP2* variants [45,46,47,48] are unable to reacetylate, with saturated stearic acid, the lyso-GPI intermediate generated by the action of PGAP3. Phospholipase D (PLD) [62], in turn, cleaves the lyso-GPI intermediate, resulting in transport of the unstable anchor and it’s attached protein, alkaline phosphatase, to the extracellular compartment.

## 4. Following Up

The syndrome, as Mabry et al. [18] reported it, remains unmistakable in presentation [63], however, our molecular genetic work identifies areas for future study. As a result of identifying the biallelic *PGAP2* variants in the Mabry syndrome index patients, we gained insight into the phenotype that became the basis of all GPIBD studies. The original case report [20] and the identification of *PGAP2* variants in these patients demonstrate the value of following patients over their lifespan, since considerable longevity is possible for patients with *PGAP2* disease [21] compared with patients known to have other likely pathogenic variants [29,30,31]. Other fruitful areas of investigation include the following.

### 4.1. Pyridoxine Responsiveness

Although Mabry et al. did not report a pyridoxine challenge when the index patients were hospitalized [20]. Our follow-up identified biallelic *PGAP2* NM_001256240.2:c.698C > T, p.Thr233Met results in the prototypical Mabry phenotype, however, we were not able to conduct a pyridoxine challenge [21]. Recent data derived from other patients suggests that the biochemical disorder associated with biallelic pathogenic variants in *PGAP2* may respond to 100 mg pyridoxine administered twice daily [64]. This work builds on foundational work that predates disease gene identification, showing that some Mabry syndrome patients respond to intravenous administration (IV) of pyridoxal hydrochloride [65]. 

The number of HPMRS phenotypes which may respond to pyridoxine, therefore, has been expanded to include *PGAP2* [61]; although methods and results suggesting that *PIGO* [66], *PIGW* [67] and *PGAP3* [68] deficiencies may be pyridoxine responsive are not consistent [69,70,71,72]. The methodology reported by Messina et al. may be useful in clarifying these results. The authors report low cerebrospinal fluid (CSF) levels of pyridoxal phosphate and 5-methyltetrahydrofolate and raised homovanillic acid may be reversed by pyridoxine and that the patient’s speech and fine motor skills improve [64].

A clinical trial may be needed to evaluate pyridoxine responsiveness in HPMRS. Interestingly, the fact that other GPIBD patients, including those with pathogenic *PIGL* [72] and *PIGS* [73] variants, may be pyridoxine responsive indicates the possible benefit of pyridoxine trials to other GPIBDs [70]. Future studies should consider standardizing the dosing, since epileptic seizures responding to pyridoxine are often reported with respect to doses of 100 mg pyridoxal hydrochloride I.V., 30 mg/kg/day pyridoxal hydrochloride orally or 50–85 mg/kg/day pyridoxal 5′phosphate (PLP) administered orally [69]. In this context, it would be useful to compare the dose, route of administration and vitamer species that produces seizure suppression in 50% of patients with various GPIBDs, as well as its impact on CSF levels of pyridoxal hydrochloride, 5-methyltetrahydrofolate and raised homovanillic acid [64].

**Figure 5:**
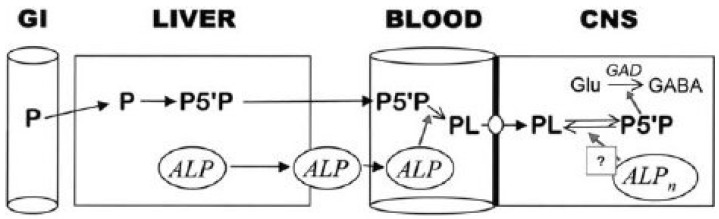
Vitamin B6 metabolism and alkaline phosphatase. Different forms of pyridoxine (P) are absorbed from the gastrointestinal tract (GI). Pyridoxine 5′phosphate (P5′P) is converted to pyridoxal (PL) in the blood, which can be transported across the blood brain barrier, where neuronal alkaline phosphatase acts to reform the metabolically active P5′P form in the CNS. Adapted from Thompson et al. [65].

The putative mechanism of pyridoxine’s anticonvulsant effect In Mabry syndrome may be as follows. Hyperphosphatasia may result in decreased availability of pyridoxal 5′phosphate pyridoxine for transport across the blood-brain barrier due to increased alkaline phosphatase. This putative insufficiency may result in decreased synthesis of the inhibitory γ amino butyric acid (GABA): resulting in seizures. Pyridoxine administration may overcome the effects of increased alkaline phosphatase and suppress seizures as GABA levels are restored [65] (Figure 5).

### 4.2. Putative Glycolipid Storage

**Figure 6:**
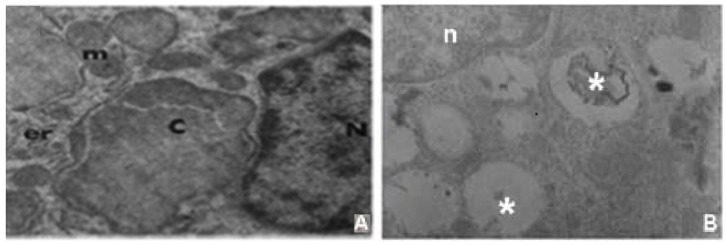
Electron micrograph of macrophages showing evidence of stored material. (**A**) Mabry syndrome index case (HPMRS3), m, mitochondria; er, endoplasmic reticulum; n, nucleus, c. stored material. Reproduced with permission from Mabry et al., J. Pediatrics; published by Elsevier, 1970. (**B**) HPMRS4 case, n, nucleus, * stored material. Adapted from Thompson et al. [28].

By contrast, it remains to be resolved if the putative glycolipid storage material reported by Mabry et al. remains stable throughout the disorder’s natural history or, for that matter, was an artifact of the tissue preparation used at the time. We suggest that if the data in the original report [20] is verified [21,28] (Figure 6), the putatively stored material might be identified using mass spectrographic analysis. It seems plausible that it may represent a portion of glycolipid comprising all or part of the glycolipid GPI anchor that is stored as a result of a possible ER-Golgi transit defect.

### 4.3. Model Organisms

If confirmed, putative glycolipid storage may provide a useful therapeutic endpoint in developing future treatments. Although transgenic GPIBD mice, such as *PIGV* Crispr-Cas9 [74] and adeno-associated virus (AVV) generated *PIGO* mice [75], are available, no storage material has been reported that might be used in preclinical work. It is likely that, even if present in some models or patients, that storage material may be associated with specific pathogenic variants or genetic backgrounds. However, with further phenotyping of HPMRS mouse models, evidence of increased ER stress causing aggregation of GPI-anchor and/or other proteins may be identified, as reported in WFS mice [76]. In the case of WFS mice, the potential of *WFS1* to delay or block neurodegeneration [76] may suggest future work in HPMRS mice; although the relative stability of HPMRS phenotypes, such as hypotonia and seizures [21], suggest divergent pathologies.

### 4.4. GPI-AP Targets

A complementary approach to identifying potential targets for interdicting GPIBD variant pathology derives from the study of GPI-AP functions. HPO analysis has suggested that 12 of the 142 APs selected for study were associated with 25 of the 34 HPO terms describing GPIBDs. Moreover, this study showed that GPI-Glypicans (GPC3 and 6) were associated with 25 of 34 HPO terms describing GPI-APs. GPC3 alone was associated with phenotypes of 6 transamidase and remodeling phenotypes. Of interest, more than one syndrome is associated with Glypican disruption, directly or indirectly. For example, Simpson-Golabi-Behmel overgrowth syndrome [77] and Saul-Wilson syndrome [78] have Glypican involvement. In particular, in Saul-Wilson syndrome, a rare skeletal dysplasia, Glypicans accumulate on fibroblasts [78]. The role of fibroblast growth factor 2 (FGFR2) in acting as a coreceptor in with Glypican for fibroblast growth factor (FGF) further supports the role of these GPI-AP interactions in development and developmental disability. FGFR2, itself, is associated with 15 of 16 HPO terms describing transamidase + remodeling. In this context, it is notable that FGFR2 itself is associated with Pfeiffer and Crouzon syndromes [79,80]. Further study of these pathways in HPMRS mouse models and human cell lines may be informative.

## 5. Conclusions

Due to advances in laboratory diagnostics as well as efforts in deep phenotyping [29,30,31,53], we are now able to offer much better genetic counselling and treatment recommendations for Mabry syndrome patients and families. Models of HPMRS1-6 will provide the means of testing future treatments for these and other GPIBDs. Together with improved diagnostics, improved treatment outcomes will increase longevity and quality of life for many patients. From the current perspective, then, the innovations made possible through the efforts of Mabry et al. to diagnose one family are relevant not only to thousands of GPIBD patients worldwide but those with developmental disabilities generally as well as other rare mendelian disorders.

## Data Availability

Not applicable.
